# Hippocampal network hyperexcitability in young transgenic mice expressing human mutant alpha-synuclein

**DOI:** 10.1016/j.nbd.2020.105226

**Published:** 2021-02

**Authors:** Clare Tweedy, Nathan Kindred, Joshua Curry, Christopher Williams, John-Paul Taylor, Peter Atkinson, Fiona Randall, Daniel Erskine, Christopheer M. Morris, Amy K. Reeve, Gavin J. Clowry, Fiona E.N. LeBeau

**Affiliations:** a Biosciences Institute, Newcastle University, Medical School, Framlington Place, Newcastle-upon-Tyne NE2 4HH, UK; b Institute of Clinical and Translational Research, Newcastle University, Medical School, Framlington Place, Newcastle-upon-Tyne NE2 4HH, UK; c Eisai Hatfield Research Laboratories, Eisai Ltd., European Knowledge Centre, Mosquito Way, Hatfield, Herts AL10 9SN, UK; d Previously Eisai AiM Institute, Eisai Inc., 4 Corporate Drive, Andover, MA 01810, USA

**Keywords:** Hippocampus, Gamma oscillations, Alpha-synuclein, Parvalbumin, Mitochondria, Burst discharges, α-syn, alpha-synuclein, ACSF, artificial cerebrospinal fluid, AD, Alzheimer's disease, ANOVA, analysis of variance, CA3, cornu ammonis 3, COX1, cytochrome *c* oxidase subunit 1, DLB, dementia with Lewy bodies, DAB, 3,3′-diaminobenzidine, EDTA, edetate disodium, EEG, electroencephalogram, Hz, Hertz, i.c., intracellular, IIDs, interictal discharges, IQR, interquartile range, KA, kainic acid, LBD, Lewy body dementia, LFP, local field potential, MCI, mild cognitive impairment, NGS, normal goat serum, NMDA, *N*-methyl-d-aspartate, NPC, no primary control, PBS, phosphate buffered saline, PBST, phosphate buffered saline tween, PD, Parkinson's disease, PDD, Parkinson's disease dementia, PDGF, platelet derived growth factor, PFA, paraformaldehyde, PV, parvalbumin, RI, rhythmicity index, RM, repeated measures, rmp, resting membrane potential, SD, standard deviation, s.e.m., standard error of the mean

## Abstract

Abnormal excitability in cortical networks has been reported in patients and animal models of Alzheimer's disease (AD), and other neurodegenerative conditions. Whether hyperexcitability is a core feature of alpha(α)-synucleinopathies, including dementia with Lewy bodies (DLB) is unclear. To assess this, we used two murine models of DLB that express either human mutant α-synuclein (α-syn) the hA30P, or human wild-type α-syn (hWT-α-syn) mice. We observed network hyperexcitability *in vitro* in young (2–5 months), pre-symptomatic transgenic α-syn mice. Interictal discharges (IIDs) were seen in the extracellular local field potential (LFP) in the hippocampus in hA30P and hWT-α-syn mice following kainate application, while only gamma frequency oscillations occurred in control mice. In addition, the concentration of the GABA_A_ receptor antagonist (gabazine) needed to evoke IIDs was lower in slices from hA30P mice compared to control mice. hA30P mice also showed increased locomotor activity in the open field test compared to control mice. Intracellular recordings from CA3 pyramidal cells showed a more depolarised resting membrane potential in hA30P mice. Quadruple immunohistochemistry for human α-syn, and the mitochondrial markers, porin and the complex IV enzyme cytochrome *c* oxidase subunit 1 (COX1) in parvalbumin (PV+)-expressing interneurons showed that 25% of PV+ cells contained human α-syn in hA30P mice. While there was no change in PV expression, COX1 expression was significantly increased in PV+ cells in hA30P mice, perhaps reflecting a compensatory change to support PV+ interneuron activity. Our findings suggest that hippocampal network hyperexcitability may be an important early consequence of α-syn-mediated impairment of neuronal/synaptic function, which occurs without any overt loss of PV interneurons. The therapeutic benefit of targeting network excitability early in the disease stage should be explored with respect to α-synucleinopathies such as DLB.

## Highlights

• Young transgenic α-syn mice exhibit network hyperexcitability in the hippocampus *in vitro*.
• Young transgenic α-syn mice have increased locomotor activity in an open field test.
• Hippocampal pyramidal cells are more depolarised in young transgenic α-syn mice.
• Increased mitochondrial cytochrome *c* oxidase (complex IV) function in PV+ interneurons in young transgenic a-syn mice.

## 1 Introduction

The Lewy body dementias (LBDs), including DLB and Parkinson's disease dementia (PDD), are together the second most common cause of neurodegenerative dementia after AD, accounting for 15–20% of dementia cases (McKeith et al., 2017). DLB is associated with progressive cognitive impairments including complex visual hallucinations, sleep disturbances as well as fluctuations in attention and cognition (McKeith et al., 2017). In DLB the cognitive symptoms occur prior to the later onset of motor symptoms, while in PDD motor dysfunction precedes any cognitive changes. Pathologically, LBDs are characterised by protein inclusions in neurons, referred to as Lewy bodies (LB) or Lewy pathology, consisting predominantly of aggregated α-syn (Spillantini et al., 1997), a synaptic protein involved in neurotransmitter release (Lautenschlager et al., 2017). In DLB aggregated α-syn is widely distributed in the brain including the hippocampus (Adamowicz et al., 2017) but how this leads to the cognitive deficits observed in LBD patients is not understood. Furthermore, it is unclear how the accumulation of α-syn aggregates impact network activity and neuronal function.

Several studies have reported changes in neuronal and network excitability linked to neurodegeneration. Patients with both mild cognitive impairment (MCI) and AD show a high co-morbidity with epilepsy (Born, 2015; Vossel et al., 2017). Furthermore, both seizures and subclinical seizures, not associated with overt motor symptoms, might drive further pathological changes (Vossel et al., 2016). Recent studies have reported that epilepsy or myoclonus (involuntary jerking of the muscles due to aberrant neuronal activity in the motor cortex) is also a feature of DLB and PDD (Morris et al., 2015; Beagle et al., 2017; Ukai et al., 2017). Numerous studies have investigated network and neuronal hyperexcitability in murine models of AD (reviewed in Busche and Konnerth, 2015; Palop and Mucke, 2016; Haberman et al., 2017), and other neurodegenerative conditions (Dougherty et al., 2014; Zhang et al., 2016). However, only two studies to-date have shown abnormal polyspike cortical activity *in vivo* in α-syn transgenic mice (Morris et al., 2015; Peters et al., 2020), but whether hyperexcitability is a consistent feature of early α-syn pathology is unclear. To address this issue we have studied mice that express human mutant α-syn (hA30P) under the control of the Thy-1 promoter, as a murine model of α-synucleinopathies such as DLB. These mice express a mutation (alanine to proline, A30P) in the SNCA gene that is associated with familial PD (Kahle et al., 2000). The A30P mutation causes increased oligomerisaton and fibrillisation of α-syn into toxic aggregates (Kahle et al., 2001). These α-syn oligomers precipitate a cascade of changes, including mitochondrial dysfunction and oxidative stress that leads to neurodegeneration (Bhat et al., 2015). hA30P mice exhibit upregulation of α-syn by one month (Kahle et al., 2000), and an age-dependent progression in α-syn pathology including increased levels of oligomers and fibrils, and phosphorylation of α-syn (Schell et al., 2009). α-syn pathology in hA30P mice causes deficits in memory function as determined using the Morris water maze and fear conditioning task (Freichel et al., 2007; Schell et al., 2009), and increasing motor dysfunction with age (Neumann et al., 2002; Ekmark-Lewen et al., 2018; Keane et al., 2019). To confirm key findings in a different α-syn transgenic model, we also tested mice that express human wild type α-syn (hWT-α-syn) under the control of the platelet-derived growth factor (PDGF) promoter (Masliah et al., 2000; Rockenstein et al., 2002). hWT-α-syn mice also exhibit cognitive dysfunction at 9 months of age with abnormal α-syn in the hippocampus and cortex (Price et al., 2010).

We have previously reported impaired mitochondrial function and cholinergically-driven fast (20–80 Hz) network gamma frequency oscillations in the hippocampus of hA30P mice aged 9–14 months (Robson et al., 2018), which corresponds to the age at which cognitive decline is reported (Freichel et al., 2007). In the current study we assessed hippocampal network oscillations evoked using the glutamate agonist kainate (KA) and found a marked hyperexcitability in the hippocampus in young mice aged 2–5 months. In addition to gamma frequency activity, interictal-like discharges (IIDs) were evident in the hippocampus *in vitro* in both α-syn transgenic mouse models. In contrast, burst-like activity was never seen in hippocampal slices taken from young control mice. We also found a lower threshold for IIDs induced by GABA_A_ receptor blockade, and a more depolarised resting membrane potential (rmp) of CA3 pyramidal cells, suggesting both neuronal and network hyperexcitability in hA30P mice. Interneurons expressing the calcium binding protein parvalbumin (PV+) are critical for normal gamma frequency oscillations (Colgin, 2016; Whittington et al., 2011), and for controlling the generation of epileptiform activity (Jiang et al., 2016). Furthermore, PV-expressing interneurons also have high energy demands (Kann et al., 2014), and express high levels of the mitochondrial enzyme cytochrome *c* oxidase (Gulyas et al., 2006), which forms complex IV of the electron transport chain. We, therefore, also investigated whether α-syn pathology specifically affected PV+ interneurons in the hippocampus of hA30P mice. Although there was no significant change in PV intensity, we did find that a proportion of PV+ cells in the hA30P mice contained human α-syn. In addition, there was an increase in the COX1/porin ratio in PV+ cells, suggestive of increased activity of complex IV in these interneurons. These findings suggest that neuronal/network hyperexcitability, associated with human mutant α-syn expression, occurs early in disease progression and could be a core feature of disease pathogenesis in conditions such as DLB.

## 2 Methods

### 2.1 Transgenic mice

Two different α-syn transgenic mouse lines were used. The first model expressed human wild type α-syn under the control of the platelet-derived growth factor (PDGF) promoter (Masliah et al., 2000). The second mouse line expressed human mutant α-syn (hA30P) under the control of the Thy-1 promoter (Kahle et al., 2000). The hA30P mice (male and female) were bred in house from homozygous breeding pairs originally supplied by Dr. P Kahle, University of Tubingen. A homozygous hA30P line was subsequently re-generated using control C57BL/6 mice from Charles River (Tranent, UK). Heterozygous offspring were genotyped (Transnetyx, Cordova, TN, USA) and new homozygous lines of both control and hA30P mice were established with the F1 offspring further genotyped. hWT-α-syn mice (Masliah et al., 2000) were provided by Dr. Birgit Hutter-Paier (QPS, Austria). Control mice were age-matched C57BL/6 either bred in house or purchased from Charles River Laboratories (Harlow, UK). Animals were housed according to ARRIVE guidelines and were maintained on a 12-h dark/light cycle with lights on at 7.00 am.

### 2.2 Slice preparation and solutions

All mice were anaesthetised with inhaled isoflurane prior to intramuscular injection of ketamine (≥ 100 mg kg^−1^) and xylazine (≥ 10 mg kg^−1^). All procedures were in accordance with the UK Animals (Scientific Procedures) Act 1986 and European Union directive 2010/63EU. When all response to noxious stimuli, such as pedal withdrawal reflex, had terminated the animals were intracardially perfused with ~25 ml of modified artificial cerebrospinal fluid (ACSF) that was composed of (mM) 252.0 sucrose, 3.0 KCl, 1.25 NaH_2_PO_4_, 24.0 NaHCO_3_, 2.0 MgSO_4_, 2.0 CaCl_2_ and 10.0 glucose. Following brain removal, 450 μm thick horizontal slices were cut using a Leica VT1000S vibratome. Hippocampal slices were then trimmed and transferred to a holding chamber at room temperature for approximately 1 h before being placed in the recording chamber where they were maintained at 32–34 °C at the interface between normal ACSF (where sucrose was replaced with 126 mM NaCl) and humidified 95% O_2_/5% CO_2_. Drugs: Kainate (25 to 200 nM, Sigma Aldrich, Gillingham, Dorset, UK); gabazine (SR-95531; 50 to 500 nM, Abcam, Cambridge, UK).

### 2.3 Recording and data acquisition

Extracellular recording electrodes were filled with normal ACSF (resistance 2–5 MΩ). Gamma frequency oscillations were evoked using the glutamatergic agonist kainate (KA) and field traces were recorded from the border between *stratum radiatum* and *stratum lacunosum moleculare* in CA3. Intracellular recordings used potassium acetate (2 M) filled glass microelectrodes (resistance 80 MΩ - 150 MΩ). Data were recorded with an Axoclamp-2B amplifier (Axon Instruments Inc., Union City, CA, USA). Extracellular data were filtered at 0.001–0.4 kHz and intracellular signals were low pass filtered at 2 kHz using Bessel filters. Mains noise was subtracted from the signal with a Humbug (Digitimer, Welwyn Garden City, Herts, UK). Data were redigitised at 10 kHz using an ITC-16 interface (Digitimer, Welwyn Garden City, UK). Data were recorded and analysed using Axograph 4.6 software (Axon Instruments Inc., Union City, CA, USA). Further offline analysis was performed using MATLAB (Mathworks, Natick, USA).

### 2.4 Data analysis of burst discharges

Bursts or single population spikes were defined as events >5 SD of baseline activity. Burst discharges were analysed by Axograph's ‘peak detection’ function for a measure of frequency (number of bursts over 60 s) and average peak-to-peak amplitude (difference between maximum and minimum positive and negative points of peak).

### 2.5 Data analysis of gamma frequency activity

Power spectra were generated using Axograph's fast Fourier transform analysis of 60 s epochs of recorded activity to give the peak frequency of the oscillations. Gamma frequency oscillations in the mouse have previously been shown to have a slower frequency compared to rat ~20–35 Hz (Robson et al., 2018), therefore, a value for area power was determined as the area under the curve in the power spectra between 15 and 45 Hz. In experiments where the KA concentration was increased incrementally every 30 mins the area power was measured over a 1 min epoch prior to the next increase in concentration (i.e min 29–30). Oscillations that were defined as “spikey” had large population events 5 x the SD of the ongoing oscillation amplitude. Rhythmicity was measured from the amplitude of the first side peak of the autocorrelation performed over 1 s traces. The rhythmicity index (RI) was on a scale of 0–1. As multiple slices can be obtained from each animal, in all the data reported below two n values (n/N) represents slice or cells, and animal numbers respectively.

### 2.6 Intracellular recordings

Sharp electrode intracellular recordings were made from presumed pyramidal cells in the CA3 region of the hippocampus. Cells were classified as pyramidal based on their documented firing properties, including spike adaption in response to a depolarising current step, and the presence of the hyperpolarisation activated current (I_h_). All intracellular recordings were made in normal ACSF (absence of synaptic blockers or KA). Resting membrane potential (rmp) (mV), firing threshold (mV), spike frequency at threshold (Hz) and spike amplitude (mV) were measured.

### 2.7 Immunohistochemistry

Two approaches were taken for preparing sections for immunohistochemistry. 1. Control and hA30P mice were anaesthetised with inhaled isoflurane prior to intramuscular injection of ketamine (≥ 100 mg kg^−1^) and xylazine (≥ 10 mg kg^−1^) and then intracardially perfused with saline followed by buffered 4% paraformaldehyde (PFA) solution. Brains were either stored in PFA or cryopreservant (glycerol and ethylene glycol in deionised water and 0.3 M PBS) and then transferred to 30% sucrose solution in phosphate buffered saline (PBS) prior to sectioning. Horizontal sections (5 μm) were cut on a cryostat and the sections allowed to air dry at 37 °C for 24 h. Sections were subjected to antigen retrieval in boiling 1 mM EDTA pH 8, for 10 min, and then rinsed in warm PBS, which was allowed to cool to room temperature. Sections were then incubated in 10% normal goat serum (NGS) (diluted in PBS with the addition of 0.1% tween 20 (PBST)) for an hour. Primary antibodies were then applied, diluted in 10% NGS PBST and incubated at 4 °C overnight. The following cocktail of antibodies was used parvalbumin (rabbit, Swant); human α-syn antibody (15G7, rat monoclonal, Enzo Life Sciences); VDAC1 (Porin, IgG2b mouse, Abcam) and mitochondrial cytochrome *c* oxidase subunit 1 (COXI, IgG2a mouse, Abcam) at dilutions of 1:1000, 1:50, 1:100 and 1:100 respectively. The following morning, after three 10 min washes in PBST the secondary antibodies were applied for 2 h at 4 °C. All secondary antibodies were diluted at 1:100 and were as follows; anti-rabbit Alexa Fluor 405, anti-rat Alexa Fluor 488, anti-mouse IgG2b Alexa Fluor 647, and anti-mouse IgG2a Alexa Fluor 546 (Thermofisher). Three 10 min PBST washes were performed, followed by a 10 min incubation with Sudan black and three subsequent 10 min PBST washes. 2. Control and hA30P mice were anaesthetised with inhaled isoflurane prior to intramuscular injection of ketamine (≥ 100 mg kg^−1^) and xylazine (≥ 10 mg kg^−1^) and cervical dislocation performed. Brains were removed and placed in formalin for 24 h, before being placed in 70% ethanol prior to processing and paraffin embedding. Formalin fixed, paraffin embedded sections (5 μm) were dewaxed in histoclear and rehydrated through a graded ethanol series to dH_2_O. Antigen retrieval was conducted at pressure in 1 mM EDTA pH 8.0. Following a dH_2_O wash, sections were blocked using 5% NGS in PBST for 1 h. Primary antibodies were then applied diluted in 5% NGS (same dilutions as outlined in method 1) and incubated for 2 h. Following 3 PBST washes secondary, fluorescence conjugated antibodies were applied for a further 2 h (same dilutions as outlined in method 1). A final 3 washes in PBST were performed before the addition of Sudan Black solution to quench autofluorescence. Sections were rinsed in PBST and dH_2_O before mounting in Prolong gold mounting media, and stored at − 20C until imaging.

Sections were imaged using a widefield imaging system, AxioImager Z2 microscope with Zen Blue software (Carl Zeiss). Images were captured of parvalbumin (PV+) positive interneurons within the CA3 region of each individual mouse, and median densitometric measurements across the neuronal area were performed. Non-specific staining intensities, as measured in no primary control images, were subtracted and measurements were normalised to unit area. We also counted the number of PV+ neurons which exhibited intense human α-syn staining above that of the surrounding neuropil.

### 2.8 Open-field activity

Mice were tested in a novel open field (4 boxes, each measuring 42 × 42 cm) to measure locomotor activity. All open field tests were performed in the morning, starting ~9.00 am with a mix of control and hA30P mice of a single sex in each testing group of 4 mice. Control and hA30P mice were further varied by randomizing the testing box they were placed in, to account for any possible variability between the positions of the four boxes in the room. The floor of each box was covered with an equal amount of sawdust, before each mouse was placed in the centre of a box and the recording started. Mice were left in a slightly darkened room without intervention for 90 min while beam breaks were recorded using the Auto-Track System of the Opto-Varimex (Columbus Instruments, USA). A measure of distance travelled (cm) was made at each minute and later grouped into 5 min blocks. The amount of time mice spent ambulatory (breaking different beams), resting (breaking no beams), and exhibiting repetitive stereotypic behaviour (breaking the same beam twice) were measured over the 90 min period.

### 2.9 Statistical analysis

Data were statistically analysed and plotted using Prism 8 (GraphPad Software, USA) and SPSS Statistics 26 (IBM, USA). Data were tested for normality (Shapiro-Wilk test) and equal variance (Brown-Forsythe test) and if the data were found to follow a normal (Gaussian) distribution then parametric tests were applied to the data. Normally distributed data were plotted as mean ± s.e.m. Data that were not normally distributed were plotted as median and interquartile range (IQR). To compare two independent samples parametric unpaired *t*-tests were used if data were normally distributed, or a non-parametric Mann-Whitney rank sum test was performed if data were not normally distributed. To compare the effect of multiple independent variables on one dependent variable, a 2-way or 3-way ANOVA was used. If comparisons were made over the same set of slices, the test also incorporated repeated measures (RM) with Greenhouse-Geisser correction if lack of sphericity was found. If an overall effect was found, multiple comparisons were performed post-hoc using Dunnett's or Tukey's tests. All tests were two-tailed and the alpha level was set at 0.05 (*p* values <0.05 indicated by * throughout). *F* and *t* distribution values are reported alongside their degrees of freedom. Fisher's exact test for small sample sizes was performed on raw data to compare observed proportions against expected proportions, with the null hypothesis being that data should be equally distributed between groups. Sex or concentration were added as stratifying layers to the analysis. For immunohistochemistry, to allow comparison of values obtained in two different sessions, data were log-transformed and presented as z-scores, calculated with comparison to that session's control mean and standard deviation. A median z-score was then calculated for each mouse.

## 3 Results

### 3.1 Increased kainate-evoked gamma network oscillations in hA30P mice

**Fig. 1:**
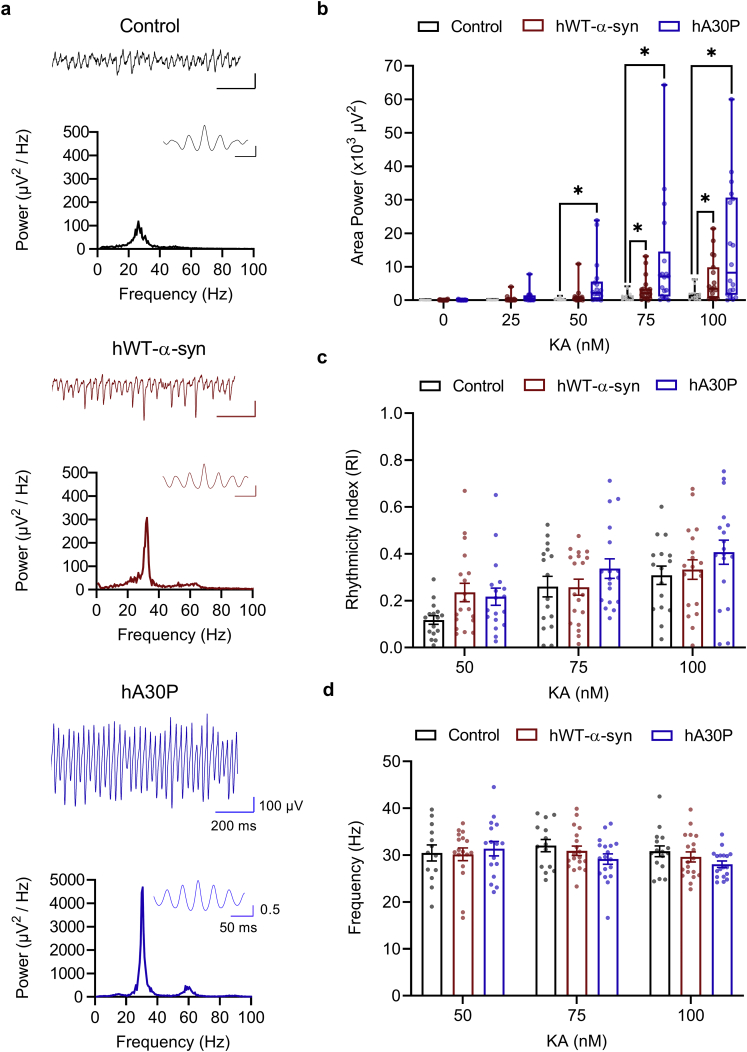
Increased KA-evoked hippocampal gamma frequency activity in slices from young α-syn transgenic mice. a) Representative traces of LFP from CA3 *st. radiatum* following bath application of 100 nM KA, power spectra and autocorrelations (inset to power spectra) in slices from young control (black), hWT-α-syn (red) and hA30P (blue) mice. Slices from α-syn transgenic mice show larger hippocampal CA3 oscillations. b) Area power within the 15–45 Hz range at increasing concentrations of KA showed a concentration x genotype interaction but no sex effect (RM 3-way ANOVA). Median and IQR area power after 30 mins at 100 nM was; Control 1335 (324–2091) μV^2^; hWTα-syn 3447 (626–9866) μV^2^; hA30P 8231 (1595–30,660) μV^2^. Asterix (*) indicates significance compared to control mice at each concentration. c) Once oscillations were established with 50 nM KA, RI increased with increasing KA concentrations regardless of genotype or sex (RM 3-way ANOVA). Mean and s.e.m. for RI after 30 mins at 100 nM was (Control 0.31 ± 0.04; hWT-α-syn 0.33 ± 0.04; hA30P 0.41 ± 0.05). d) Peak frequency (15–45 Hz) once oscillations were established from 50 nM KA onwards, showed no effect of sex, genotype, or KA concentration on oscillation frequency. Mean and s.e.m. for frequency after 30 mins at 100 nM was (Control 30.8 ± 1.1 Hz; hWT-α-syn 29.6 ± 1.1 Hz; hA30P 28.1 ± 0.7 Hz). Control *n* = 8 slices/4 male mice, 8 slices/4 female mice; hWT-α-syn *n* = 12 slices/4 male mice, 7 slices/2 female mice; hA30P-α-syn *n* = 9 slices/5 male mice, 9 slices/4 female mice (Some slices at 50–75 nM excluded as defined peak in spectrum not yet evident).

Transgenic α-syn mice exhibit both cognitive dysfunction and marked motor impairments from ~9–12 months of age (Masliah et al., 2000; Neumann et al., 2002; Freichel et al., 2007; Schell et al., 2009; Price et al., 2010), but whether early prodromal changes are evident is currently unclear. To assess the impact of abnormal human α-syn expression on hippocampal network activity in young, pre-symptomatic mice, we compared two transgenic α-syn mouse lines (see Methods), the hA30P and hWT-α-syn, with control mice (Kahle et al., 2000; Masliah et al., 2000). The glutamatergic agonist kainate (KA, 25–150 nM) evokes robust network gamma frequency oscillations in hippocampal slices taken from young control mice (Fisahn et al., 2004). To determine whether the expression of abnormal α-syn altered the generation of oscillations we first applied KA (Fig. 1) at increasing concentrations of 25 nM (25–100 nM). Recordings were made in *st. radiatum* in CA3 of the hippocampus from control mice aged 2–5 months and age-matched hA30P and hWT-α-syn mice (Fig. 1a).

Upon incrementally increasing the KA concentration by 25 nM every 30 min, gamma frequency activity developed and increased in area power in all genotypes and sexes (*F* (4, 186.51) = 19.49, *p* = 0.0001; mixed RM 3-way ANOVA). At concentrations up to 100 nM, only network gamma activity was observed (see below), therefore, we initially analysed the properties of this network oscillation at concentrations from 25 to 100 nM KA. Analysis of area power (Fig. 1b) indicates a genotype x concentration interaction (*F* (8, 186.48) = 5.71, *p* = 0.0001; mixed RM 3-way ANOVA). Post-hoc comparisons (Dunnett's test) showed that slices from hA30P mice exhibited a significantly greater gamma oscillation power than slices from control mice at 50 nM (*p* = 0.025), 75 nM (*p* = 0.026), and 100 nM KA (*p* = 0.0061). Slices from hWT-α-syn mice also showed increased KA-induced gamma oscillation area power, compared to slices from control mice at 75 nM (*p* = 0.045) and 100 nM KA (*p* = 0.032). No effect of sex was found over increasing KA concentrations (*F* (4, 186.51) = 0.32, *p* = 0.86; mixed 3-way RM ANOVA) or between genotypes (*F* (2, 47.28) = 0.61, *p* = 0.55; mixed 3-way RM ANOVA).

KA-induced gamma frequency oscillations were evident in the recordings in the majority of slices at 50 nM KA. We therefore compared the rhythmicity index (RI) values and peak frequency (see Methods) across the genotypes from 50 to 100 nM KA. The RI significantly increased (Fig. 1c) with increasing concentrations of KA regardless of genotype or sex (*F* (2, 94) = 20.66, *p* = 0.0001; RM 3-way ANOVA). There was, however, no significant difference in RI values between mouse genotype (*F* (2, 47) = 2.43, *p* = 0.10; RM 3-way ANOVA) or sex (*F* (1, 47) = 0.43, *p* = 0.52; RM 3-way ANOVA). Peak frequency of the oscillation did not change significantly over increasing KA concentrations (Fig. 1d) once oscillations were established at 50 nM KA (*F* (2, 132) = 1.47, *p* = 0.23; 3-way ANOVA), and there was no significant effect of sex (*F* (1, 132) = 0.83, *p* = 0.36; 3-way ANOVA) or genotype (*F* (2, 132) = 1.10, *p* = 3.34; 3-way ANOVA) on oscillation frequency (Fig. 1d).

### 3.2 Hippocampal slices from transgenic α-syn mice show network hyperexcitability

**Fig. 2:**
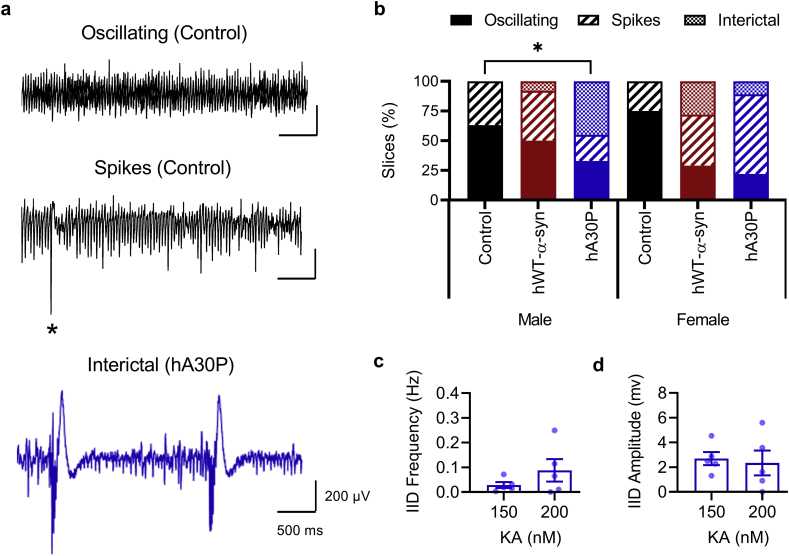
Hippocampal CA3 network hyperexcitability is evident in slices from young transgenic α-syn mice. a) Representative LFP traces from CA3 *st. radiatum* following bath applications of 150 nM KA. Slices from control mice showed either normal gamma frequency oscillations or spikey oscillatory activity (asterix *) at 150 nM KA. In some slices from both transgenic α-syn mouse lines occasional IIDs occurred during the on-going gamma activity. b) Proportion (%) of slices exhibiting each type of activity for control (black), hWT-α-syn (red) and hA30P (blue) mice, separated by sex. Solid colour bar indicates oscillating slices, hashed bar indicates spikey activity, and dotted bar indicates IID activity. Comparing the distribution of activity showed no sex or genotype effects (Fisher's exac*t-*test) but an increase in IIDs in the male hA30P mouse group compared to control mice. c) Mean burst frequency 150 nM KA = 0.03 ± 0.01 Hz, 200 nM KA = 0.09 ± 0.05 Hz; *t* (4) = 1.752, *p* = 0.16; paired *t-*test, two-tailed. d) Mean burst amplitude 150 nM KA = 2.7 ± 0.5 mV, 200 nM KA = 2.3 ± 1.0 mV; *t* (4) = 0.562, *p* = 0.604; paired *t-*test, two-tailed.

As the concentration of KA was increased further to 150 nM, oscillations with large population events we termed “spikey” (see Methods) occurred in slices from all genotypes (Fig. 2a), possibly reflecting a shift to a more excitable state. Statistically there were no differences in the distribution of slices with spikey oscillations between each genotype (*p* = 0.78, Fisher's exact-test value 0.73). However, interestingly, a subset (average 21.6%) of slices from transgenic α-syn mice exhibited regular interictal discharges (IIDs) at 150 nM KA, interspersed within the gamma frequency activity (Fig. 2a). IIDs were observed in slices from both hA30P mice and hWT-α-syn mice, but never in slices from control mice (Fig. 2b). Furthermore, no IIDs were seen in any slices before the application of KA in any genotype. Interestingly, the incidence of IIDs appeared to be more prevalent in slices from male hA30P mice (Fig. 2b). A statistical comparison of the distribution of IIDs by genotype and sex revealed a significant difference in the slices from male mice (*p* = 0.042, Fisher's exact value 5.55), but not the female mice (*p* = 0.26, Fisher's exact-test value 2.45). Post-hoc comparisons indicated there were more IIDs occurring in slices from male hA30P compared to male control mice (*p* = 0.016, Fisher's exact-test value 4.65). Slices from male hWT-α-syn mice also exhibited IIDs in the presence of 150 nM KA but, although male control mice had no IIDs, there was no statistically significant difference (*p* = 0.20, Fisher's exact-test value 0.70) between male hWT-α-syn and male control mice.

In slices from male and female hA30P mice at 150 nM KA, IIDs had an average frequency of 0.03 Hz (± 0.01; Fig. 2c) and an average amplitude of 2.7 mV (± 0.5; Fig. 2d). When the concentration of KA was increased again to 200 nM, neither IID frequency (*t* (4) = 1.752, *p* = 0.16; paired *t* test, two-tailed) or amplitude (*t* (4) = 0.562, *p* = 0.604; paired *t* test, two-tailed) changed significantly (Fig. 2c,d). Slices from control mice still showed no IID activity at 200 nM KA.

### 3.3 Young hA30P mice have increased locomotor activity

**Fig. 3:**
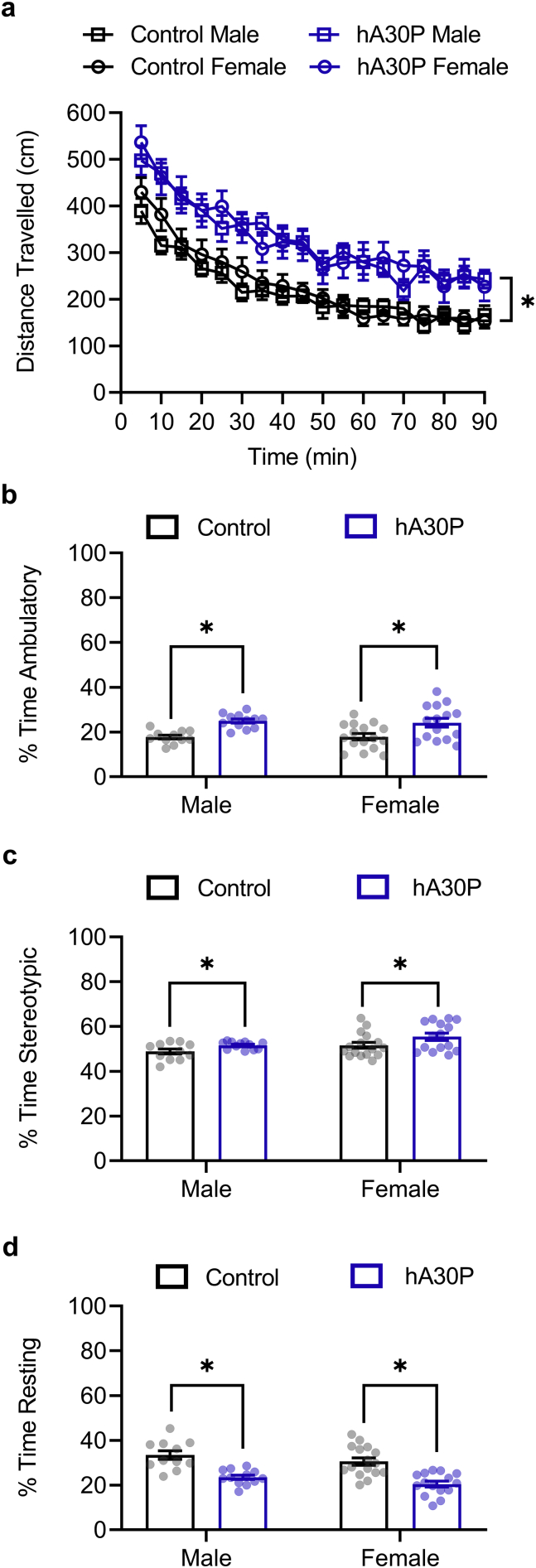
hA30P mice exhibit increased locomotor activity in the open field test. a) Distance travelled over 90 min in open field, with data in 5 min bins in control (black) and hA30P (blue) mice, separated by sex (squares = male, circles = female). RM 3-way ANOVA showed an effect of time and genotype overall. b) Percentage of time mice spent ambulatory, (c) exhibiting repetitive stereotypic behaviour, and (d) resting in control (black) and hA30P (blue) mice, separated by sex. 2-way ANOVAs of genotype/sex showed hA30P mice spending significantly more time ambulatory and exhibiting stereotypic behaviour, but less time resting. Female mice also spent more time than males exhibiting stereotypic activity regardless of genotype. Control mice *n* = 11 males, 16 females. hA30P mice n = 12 males, 15 females.

Several studies have also suggested that cortical hyperexcitability might be associated with hyperactivity (Procaccini et al., 2013; Hall et al., 2015). Hyperactivity has been reported in older hA30P (Freichel et al., 2007) and hA53T-α-syn mice (Unger et al., 2006), but there are no reports of locomotor behaviour in mice as young as 2 months of age. We, therefore, measured locomotor and stereotypic activity using an open field test (Fig. 3) in 2–5 month old hA30P mice and control mice. In view of the sex difference (outlined above) showing a greater prevalence of IIDs in male hA30P mice, we performed a RM 3-way ANOVA which showed a significant effect of time (*F* (7.50, 374.90) = 91.93, *p* = 0.0001; 3-way RM ANOVA) and genotype (*F* (1, 50) = 20.70, *p* = 0.0001; 3-way RM ANOVA), but not sex (*F* (1, 50) = 0.11, *p* = 0.74; 3-way RM ANOVA) on the distance travelled in the open field test (Fig. 3a). Both control and hA30P mice habituated to the novel environment (Fig. 3a), showing decreased activity levels at 90 min compared to 5 min (*p* = 0.0001, Tukey's test). Activity levels plateaued from 50 min with no further significant decrease up to 90 min (*p* = 0.20, Tukey's test).

The proportion of time mice spent ambulatory (Fig. 3b) was also significantly greater in hA30P mice than control mice (*F* (1, 50) = 19.88, *p* = 0.0001; 2-way ANOVA), as was the proportion of time spent exhibiting repetitive stereotypic (Fig. 3c) behaviour (*F* (1, 50) = 6.0829, *p* = 0.017; 2-way ANOVA). Female mice, regardless of genotype, appeared to exhibit more stereotypic behaviour than male mice (*F* (1, 50) = 6.0831, *p* = 0.017; 2-way ANOVA). As a result of the increased activity levels, hA30P mice spent a smaller proportion of time resting (Fig. 3d) than control mice (*F* (1, 50) = 42.67, *p* = 0.0001; 2-way ANOVA) so overall hA30P mice demonstrate significant hyperactivity.

### 3.4 Decreased threshold for gabazine-evoked burst discharges in hA30P mice

**Fig. 4:**
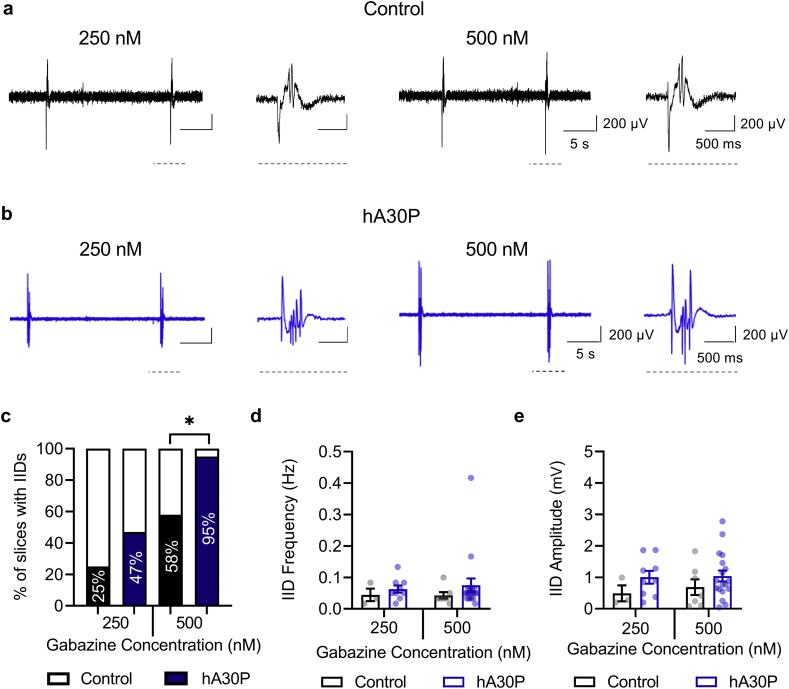
α-syn over-expression lowers the threshold for gabazine-evoked burst discharges in the hippocampus in hA30P mice. Representative extracellular field traces from CA3 showing IIDs at 250 nM gabazine and 500 nM gabazine in (a) control and (b) hA30P mice, with last discharge in each trace expanded. Gabazine was increased incrementally by 50 nM until 500 nM, at 20 min intervals for a total application time of 3 h 20 mins. c) Percentage of slices with IIDs at 250 nM and 500 nM gabazine in control (black) and hA30P mice (blue), analysed by Fisher's exact test which revealed no sex effect (so data combined) and a significant genotype effect specifically at 500 nM (* indicates significance). d) Frequency and (e) amplitude of IIDs in control (black) and hA30P mice (blue) showed no significant differences (2-way ANOVA). Control *n* = 6 slices/3 male mice, 6 slices/3 female mice; hA30P n = 8 slices/4 male mice, 11 slices/4 female mice.

To determine if the hyperexcitability observed in hA30P mice, outlined above, was specific to increasing excitatory drive with KA, or could also be revealed with reduced inhibition, we used the selective GABA_A_ receptor antagonist gabazine (SR-95531). Blockade of GABA_A_ receptors in the hippocampus with gabazine evokes IIDs at concentrations of ~5 μM *in vitro* (Behrens et al., 2007). To determine if there are changes in the threshold for IID induction in slices from hA30P mice we started with a very low concentration of gabazine (50 nM), and increased incrementally (by 50 nM until 500 nM), at 20 min intervals. Very few slices from either control or hA30P mice showed any IID activity until a concentration of ~250 nM gabazine after which large burst discharges in the LFP could be seen (Fig. 4a,b). We calculated the proportion of slices from control and hA30P mice showing IIDs at two sub-maximal concentrations of gabazine: 250 nM and 500 nM (Fig. 4c). At 500 nM gabazine, the distribution was statistically significant (*p* = 0.022, Fisher's exac*t-*test value 6.24) with more IIDs in the slices from hA30P (Fig. 4c) mice regardless of sex.

To compare IID frequency (Fig. 4d) and amplitude (Fig. 4e) at 250 and 500 nM gabazine we only included slices where at least one IID occurred during the recording. Due to the low sample size (with zero IID data excluded), and the lack of sex effect in the proportion of slices with IIDs, data were combined from male and female mice. Neither the frequency (*F* (1,33) = 0.84, *p* = 0.36; 2-way ANOVA), nor the amplitude (*F* (1, 33) = 2.51, *p* = 0.12; 2-way ANOVA), of IIDs differed in hA30P compared to control mice. Thus, once established, IID frequency and amplitude were similar between control and hA30P mice, although the slices from hA30P mice were more likely to shift to IID activity at the lower gabazine concentrations.

### 3.5 CA3 pyramidal cells from hA30P mice are more depolarised with higher firing rates

**Fig. 5:**
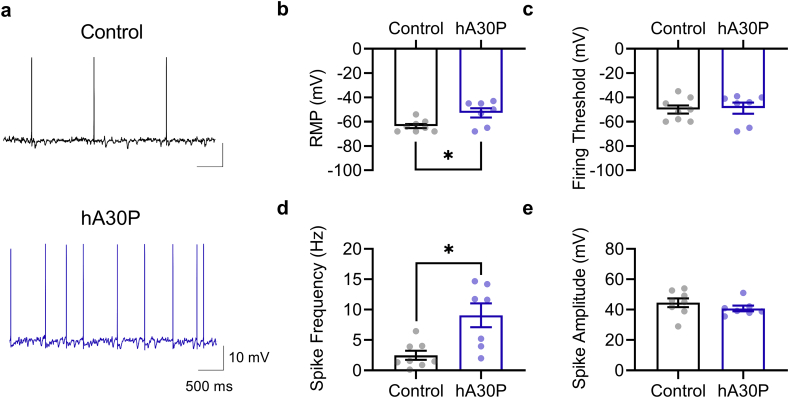
Hippocampal CA3 pyramidal cells are more depolarised in young hA30P mice. a) Representative intracellular pyramidal cell recording at firing threshold in control (black) and hA30P (blue) mice. Plots show (b) resting membrane potential (rmp), (c) firing threshold, (d) spike frequency, and (e) spike amplitude in control (black) and hA30P (blue) mice. Each parameter measured by unpaired *t-*tests and significance is marked by * for *p* < 0.05. Control n = 8 cells from 2 male and 4 female mice and hA30P *n* = 7 cells from 5 male and 2 female mice.

To assess the excitability of CA3 pyramidal cells intracellular recordings were made in control and hA30P mice in the absence of KA (Fig. 5). A comparison of rmp values (Fig. 5b) showed a significant effect of genotype (*t* (13) = 2.73, *p* = 0.017; unpaired *t-*test, two-tailed). The mean rmp for control cells was −63.6 mV ± 1.7 (n/*N* = 8 cells/6 mice) and for hA30P cells was −52.7 mV ± 3.8 (7 cells/7 mice). Firing threshold was not different (*t* (13) = 0.20, *p* = 0.84; unpaired *t-*test, two-tailed) between groups (Fig. 5c) but a comparison of spike frequency at firing threshold (Fig. 5d) revealed that hA30P mice exhibited higher spike frequencies (*t* (13) = 3.29, *p* = 0.006; unpaired *t-*test, two-tailed). However, spike amplitude (Fig. 5e) was not significantly different (*t* (13) = 1.07, *p* = 0.31; unpaired *t-*test, two-tailed). These data demonstrate that pyramidal cells from hA30P mice had a more depolarised rmp and a higher firing frequency at threshold, which could have contributed to the greater power of KA oscillations, increased incidence of IIDs evoked by KA, and the lower threshold for gabazine-evoked IIDs.

### 3.6 α-syn and mitochondrial function in PV+ interneurons in hA30P mice

**Fig. 6:**
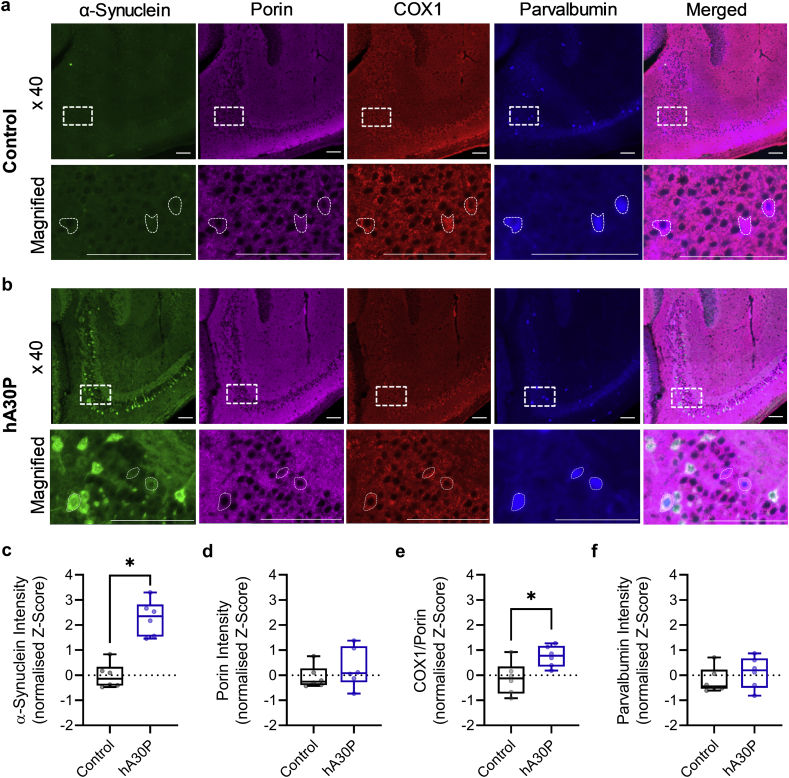
α-syn and mitochondrial markers in PV+ cells from young hA30P mice. Representative images showing quadruple immunohistochemical staining for human α-syn, porin, COXI, PV and the merged image in hippocampal CA3 sections from (a) male control and (b) hA30P mice. Boxed areas in x 40 images are shown expanded in the magnified images below. c) Human α-syn intensity was significantly higher in PV+ cells from hA30P mice compared to control mice. PV+ cells in hA30P mice were further classified depending upon whether there was colocalisation of human α-syn (+) or not α-syn (−). Magnified images (Fig. 6b) show one PV+ cell co-labelled with α-syn and two PV+ cells that did not strongly stain for human α-syn. Overall there was no significant change in porin intensity between control and hA30P mice (d). However, there was an increase in the (e) COX1/porin ratio but no change in PV intensity (f) between control and hA30P mice. Control n = 6 male mice (9–42 cells per mouse), hA30P n = 6 male mice (17–33 cells per mouse). Scale bars = 100 μm.

Normal mitochondrial function within PV+ interneurons is critical for maintaining the correct excitatory/inhibitory balance for the generation of gamma frequency oscillations in a network (Kann et al., 2011; Whittaker et al., 2011; Kann et al., 2014). However, aggregated α-syn inhibits mitochondrial function (Bhat et al., 2015; Reeve et al., 2015; Ludtmann et al., 2018), and we have previously reported an age-dependent reduction in mitochondrial complex IV expression in hA30P mice (Robson et al., 2018). We, therefore, wondered whether an early PV+ cell mitochondrial dysfunction could potentially underlie the hyperexcitability observed in the hA30P mice. We performed quadruple immunohistochemistry for PV, α-syn, and the mitochondrial markers porin and COX1 in male control and hA30P mice (Fig. 6a,b). COX1 is the catalytic subunit 1 of cytochrome *c* oxidase and is a viable marker of complex IV activity. Porin measures total mitochondria mass, while the COX1/porin ratio accounts for possible changes in mitochondrial number, and so provides a more accurate measure of mitochondrial respiratory chain complex function. To determine whether human α-syn was expressed in PV+ cells in young hA30P mice we first measured the intensity of human α-syn expression. As expected, α-syn immunofluorescence in PV+ cells in control mice was only at background levels (Fig. 6a) while human α-syn staining was evident in some PV+ cells in hA30P mice (Fig. 6b). The median intensity of human α-syn immunostaining in PV+ interneurons in hA30P mice was significantly greater than control mice (Fig. 6c; *U* = 0, *p* = 0.0022; Mann-Whitney test). Quantification showed that human α-syn was present in 25.4 ± 3.7% of PV+ cells counted in CA3 in hA30P mice. There was no significant difference in the expression of porin between mouse genotypes (Fig. 6d; *U* = 11, *p* = 0.31; Mann-Whitney test). However, there was an increase in the ratio of COX1/porin expression between control and hA30P mice that was statistically significant (Fig. 6e; *U* = 4, *p* = 0.026; Mann-Whitney test). Importantly, there was no significant difference in PV expression between control and hA30P mice (Fig. 6f; *U* = 12, *p* = 0.39; Mann-Whitney test). These data show that a significant proportion of PV+ cells in hA30P mice express human α-syn and exhibit increased complex IV activity as measured by COX1 immunohistochemistry.

## 4 Discussion

We have found that in young, pre-symptomatic mice expressing either hWTα-syn or mutant A30P α-syn, low concentrations of KA elicited significantly larger gamma frequency oscillations, while at higher concentrations a proportion of slices exhibited regular IIDs. Interestingly, in the hA30P mice this increased excitability was more prevalent in slices from male mice. In marked contrast to both α-syn transgenic mice lines, no slices from age-matched control mice exhibited IIDs at these low KA concentrations. Slices from hA30P mice also showed a reduced threshold for generating interictal activity due to blockade of GABA_A_ receptors with gabazine. In addition, CA3 pyramidal cells had a more depolarised resting membrane potential and higher firing frequency. These changes in neuronal and network excitability were accompanied by hyperlocomotion in the open field test in hA30P mice. As well as changes in excitation, this shift to a more excitable hippocampal network in the young mice expressing human mutant α-syn, could reflect impaired inhibitory function. In the young hA30P mice we found evidence of human mutant α-syn in the soma of ~25% of PV+ cells in the hippocampus. Although PV intensity was unchanged, there was an increase in the COX1/porin ratio in PV+ cells, suggesting an early increase in activity of complex IV of the mitochondrial respiratory chain in hA30P mice.

The changes in network excitability we observed in the α-syn transgenic mice at 2–5 months of age occur long before the reported cognitive dysfunctions in either line (Freichel et al., 2007; Price et al., 2010), but are consistent with *in vivo* sleep-related oscillation changes we have recently reported in young hA30P mice (Stylianou et al., 2020). In hA30P mice pathological α-syn (phosphorylated at serine 129) is present in the hippocampus pre-symptomatically and increases with age (Schell et al., 2009; Fagerqvist et al., 2013). However, despite this early pathology, cognitive deficits, including impaired performance in the Morris water maze, impaired fear conditioning and active avoidance behaviour, are not evident in hA30P mice until 12 months of age (Freichel et al., 2007). Similarly, in hWTα-syn mice cognitive deficits assessed using the Morris water maze occurred at 9 months (Price et al., 2010). These data suggest that there needs to be an age-dependent accumulation of pathology before measurable cognitive changes occur in either the hA30P or hWTα-syn mice. The Thy-1 promoter targets a more widespread neuronal population than the PDGF promoter leading to higher levels of α-syn (Rockenstein et al., 2002), which could explain the greater incidence of IIDs in hA30P mice compared to hWT-α-syn mice. Both mouse strains exhibit α-syn pathology from 2 months of age (Masliah et al., 2000; Kahle et al., 2000) which, although not yet resulting in cognitive dysfunction, could still be sufficient to impair the normal excitatory/inhibitory balance. Consistent with this presumed CA3 pyramidal cells were more depolarised, with higher firing rates in the hA30P mice compared to control mice.

We have previously shown reduced cholinergic-driven (carbachol) gamma frequency activity in the hippocampus *in vitro* in older (9–16 months) hA30P mice, but oscillations were normal at 2–6 months of age (Robson et al., 2018). In the current study, using KA to evoke oscillations, the increased excitability in the young hA30P mice became evident with the appearance of IIDs at the higher (150 nM) KA concentrations, suggesting that only with a strong excitatory drive does the network shift into an abnormal, hyperexcitable state in the hA30P mice. Alternatively, we cannot exclude the possibility that the neuronal populations that become activated by KA *versus* carbachol are different, and thus could be differently affected by α-syn. Consistent with the idea of strong excitation unmasking network hyperexcitability, one study reported that excess α-syn impaired synaptic vesicle recycling, but only during intense stimulation (Busch et al., 2014), supporting the view that α-syn is critical for maintaining synaptic function during repetitive stimulation (Lautenschlager et al., 2017).

Our result showing network hyperexcitability *in vitro* in both α-syn transgenic mouse lines are consistent with two previous studies that showed abnormal polyspikes in cortical EEG recordings in awake-freely moving α-syn transgenic mice (Morris et al., 2015; Peters et al., 2020). Indirect evidence of hyperexcitability with hippocampal circuit remodelling has been reported in A53T-α-syn mice (Singh et al., 2019), and one recent study suggests that this also precedes cognitive decline (Peters et al., 2020). We have now found that hyperexcitability is also evident *in vitro* and can occur at an early stage of disease progression, before the onset of cognitive or motor symptoms in both mutant hA30P α-syn and human hWT-α-syn mice. Toxic α-syn oligomers form pores in the neuronal plasma membrane, allowing calcium entry, and a subsequent increase in synaptic transmission (Pacheco et al., 2015). Increased glutamate release and hyperexcitability could thus be an early consequence of α-syn pathology in the transgenic mice. α-syn release into the extracellular space is also activity dependent (Yamada and Iwatsubo, 2018), so increased activity levels in the hippocampus could potentially drive further α-syn release. We also showed that slices from hA30P mice were more likely to exhibit burst discharges at lower levels of GABA_A_ receptor blockade with gabazine than control mice. This altered GABA_A_ receptor sensitivity in hA30P mice is similar to data *in vivo* using AD transgenic mice (Palop et al., 2007) and suggests impaired inhibition may also be present in hA30P mice. Overall subtle shifts in the excitatory/inhibitory balance led to the generation of IIDs at the higher KA concentrations in the transgenic α-syn mice.

Although no sex differences in the expression levels of α-syn were reported for the hA30P mice (Kahle et al., 2000), we found a greater prevalence of IIDs in slices from male mice. A recent report using A53T transgenic mice also showed female mice had less neurodegeneration and neuroinflammation associated with milder motor and non-motor deficits than male mice (Costa et al., 2020). There is also an increased incidence of epilepsy in male rodents (Scharfman and MacLusky, 2014), but the reasons for these differences in both humans and rodents remains to be determined. Interestingly, myoclonus and male gender are associated with a lower age of onset of cognitive impairments in DLB patients (Morris et al., 2015), again suggesting that hyperexcitability might exacerbate the spread of α-syn pathology. In humans increased excitability in the hippocampal CA3 region and dentate gyrus is associated with impaired memory function (Yassa et al., 2010). In two clinical studies patients with MCI had increased hippocampal activity and cognitive deficits, that were reversed by treatment with the anti-epileptic agent levetiracetam (LEV) (Bakker et al., 2012). Consistent with this in transgenic AD mice, a reduction of hyperexcitability with LEV also reduced excitability, and improved memory performance (Sanchez et al., 2012; Hall et al., 2015), possibly *via* changes in neurogenesis (Fu et al., 2019). It is currently unclear whether similar cortical hyperexcitabilty occurs in pre-symptomatic patients that progress to DLB. However, the early increase in hyperexcitabilty we have observed suggests excess network excitation may in fact be an important feature of α-syn pathology.

Although the mechanisms are unclear previous studies have linked hyperlocomotion in the open field test and hippocampal hyperexcitability (Procaccini et al., 2013; Yoshikawa et al., 2018). We also found a significant increase in the distance travelled in both male and female hA30P mice at 2–5 months of age. Our results are consistent with a previous study showing a significant increase in distance travelled over the first 30 mins of the test in hA30P mice aged 4 months, which became increasingly significant at 8 months of age (Freichel et al., 2007). Hyperlocomotion in the open field test has been reported in a number of transgenic models of neurodegeneration including AD (Sanchez et al., 2012; Hall et al., 2015) and Huntington's disease (Dougherty et al., 2014) which also exhibit hyperexcitability. Treatment of hyperexcitability in AD mice with LEV, in addition to improving cognitive function, also reduced the hyperlocomotion (Sanchez et al., 2012; Hall et al., 2015). However, we cannot confirm a direct association between hyperexcitability and hyperlocomotion and we also cannot exclude the possibility that changes to the dopaminergic system in the hA30P mice might also contribute to hyperactivity, as found in A53T transgenic mice (Unger et al., 2006), as dopamine neurons in substantia nigra are reduced in older hA30P mice (Keane et al., 2019).

In the young h30P mice we did not find any reduction in PV expression. We have previously shown that end-stage DLB is associated with a loss of PV+ cells in the CA3 region of the hippocampus in human post-mortem tissue (Bernstein et al., 2011), while others have found no change in PV+ cell numbers in the superior temporal sulcus (Gomez-Tortosa et al., 2001). The reason for these discrepancies are unclear, but may depend upon the region studied, or disease stage. It is possible that loss of PV+ cells in hA30P mice may occur later in the disease progression, when we know gamma frequency activity is impaired (Robson et al., 2018). Therefore, further studies in aged hA30P mice are needed to determine whether there is a loss of hippocampal PV+ cells in this model of DLB. There are also considerable differences reported in the literature with respect to the proportion of interneurons containing intracellular aggregates of α-syn. We have previously shown that in post-mortem tissue from patients with DLB only ~2–4% of PV+ cells in the hippocampus exhibited α-syn expression (Bernstein et al., 2011), while in the hippocampus of PD patients >40% of PV+ cells had intracellular α-syn (Flores-Cuadrado et al., 2016). In the hA30P mice we found 25% of PV interneurons contained human mutant α-syn. In hA30P mice α-syn expression is driven by the Thy-1 promoter and detailed gene transcription studies suggested that Thy-1, although a pan-neuronal marker, is predominantly expressed in pyramidal cells in the cortex and hippocampus (Sugino et al., 2006). In a different transgenic mouse strain (hA53T), where expression was also driven by the Thy-1 promoter, approximately 8–10% of PV+ cells expressed human mutant wild type α-syn (Martin et al., 2014). It is possible that in our study with hA30P mice interneurons could also be dysfunctional. One study using the hWTα-syn transgenic mouse model reported complex bursts of spontaneous inhibitory post-synaptic currents recorded in cortical pyramidal cells at three months of age (Wu et al., 2010). Future studies need to assess PV cell activity in hA30P mice but also other interneuron populations such as somatostatin-expressing cells, which were found to be hyperactive in a murine model of frontotemporal dementia (Zhang et al., 2016).

Our previous study (Robson et al., 2018) showed decreased global expression for complex IV, suggesting reduced mitochondrial activity in the aged hA30P mice. These findings are consistent with other studies in older α-syn transgenic mice which demonstrate impaired mitochondrial function (Martin et al., 2014). However, in the current study we found an increase in COX1/porin ratio in PV+ cells in the hA30P mice. This could reflect an increase in mitochondrial respiratory chain activity in young hA30P mice, perhaps as a result of increased activity in these interneurons in an attempt to constrain hyperexcitability in the network. Alternatively, complex IV activity may be upregulated at this early stage of α-syn pathology as a consequence of changes in other respiratory chain complexes or perhaps even as a direct consequence of α-syn pathology. Overall our data demonstrate that detailed studies of mitochondrial function are required at this early stage of disease.

Although there is considerable evidence for co-morbidity of AD and epilepsy (Vossel et al., 2017) there is now growing evidence to suggest abnormal cortical activity in patients with LBD. One of the best established EEG findings in LBDs is the presence of transient sharp-waves, predominantly in posterior derivations (Briel et al., 1999), that are used as a supportive feature for diagnosis (McKeith et al., 2017). A recent investigation of the incidence of epilepsy-related manifestations found that DLB patients had an 11.5% cumulative probability of developing seizures, but DLB patients were more likely to develop myoclonus, with a probability of 58.1% (Beagle et al., 2017). Interestingly there are also reports in the literature of the similarity in clinical presentation for patients with epilepsy and DLB (Park et al., 2014; Ukai et al., 2017). Under-diagnosis of epilepsy or hyperexcitability in AD is thought to occur and silent, sub-clinical seizures during sleep have been identified in AD (Lam et al., 2017). We propose that hyperexcitability is also likely to be under-diagnosed in LBDs. Furthermore, as there is growing interest in understanding prodromal DLB (McKeith et al., 2020), our data suggest that hyperexcitability may be a potential early biomarker of pathology.

## 5 Conclusion

The presence of early network hyperexcitability may have profound effects on the progression of pathology as the abnormal aggregation of α-syn is known to be activity dependent (Lautenschlager et al., 2017). Our data suggest that hippocampal hyperexcitability occurs long before overt cognitive dysfunction in the hA30P mice and could, therefore, be a driver of further pathological damage. Hyperexcitability is not thought to be compensatory, as pharmacological reduction with LEV improved cognition in both patients (Bakker et al., 2012; Bakker et al., 2015), and murine models of dementia (Sanchez et al., 2012; Haberman et al., 2017). Targeted intervention to reduce abnormal network hyperactivity might provide a therapeutic strategy to delay the onset of later neurodegenerative changes which, translated into the clinic, could postpone the onset of cognitive decline by many years in patients. Our data shows that DLB patients might also benefit from improved, early detection of abnormal excitability, and interventions aimed at reducing abnormal neuronal activity.

## Funding

10.13039/501100000265Medical Research Council CASE award MR/L015528/1 to FLB and GC.

## CRediT authorship contribution statement

CT, NK, JC, CW, AR and FENL investigation; CT, NK, AR formal analysis; CT and AR visualisation; CT, AR, DE, GC, JPT, CMM and FENL edited and revised manuscript; CT, AR, GC and FENL review and editing; FR, GC and FENL supervision; CT, AR, GC, and FENL conceptualisation; FENL writing; PA, FR, GC and FENL funding acquisition.

## Declaration of Competing Interest

FENL and GJC were funded by a Medical Research Council CASE studentship with Eisai (PA and FR) awarded to CT. FENL is also an unpaid advisor for Neurexpert Ltd.

J-PT has acted as a consultant to Heptares-Sosei, Kyowa-Kirin and received speaker fees from GE Healthcare.

CT, NK, JC and CW declare no conflict of interest

## References

[bb0005] Adamowicz D.H., Roy S., Salmon D.P., Galasko D.R., Hansen L.A., Masliah E., Gage F.H. (2017). Hippocampal alpha-Synuclein in dementia with Lewy bodies contributes to memory impairment and is consistent with spread of pathology. J. Neurosci..

[bb0010] Bakker A., Krauss G.L., Albert M.S., Speck C.L., Jones L.R., Stark C.E., Yassa M.A., Bassett S.S., Shelton A.L., Gallagher M. (2012). Reduction of hippocampal hyperactivity improves cognition in amnestic mild cognitive impairment. Neuron.

[bb0015] Bakker A., Albert M.S., Krauss G., Speck C.L., Gallagher M. (2015). Response of the medial temporal lobe network in amnestic mild cognitive impairment to therapeutic intervention assessed by fMRI and memory task performance. Neuroimage Clin..

[bb0020] Beagle A.J., Darwish S.M., Ranasinghe K.G., La A.L., Karageorgiou E., Vossel K.A. (2017). Relative incidence of seizures and myoclonus in Alzheimer’s disease, dementia with Lewy bodies, and frontotemporal dementia. J. Alzheimers Dis..

[bb0025] Behrens C.J., van den Boom L.P., Heinemann U. (2007). Effects of the GABA(A) receptor antagonists bicuculline and gabazine on stimulus-induced sharp wave-ripple complexes in adult rat hippocampus in vitro. Eur. J. Neurosci..

[bb0030] Bernstein H.G., Johnson M., Perry R.H., LeBeau F.E., Dobrowolny H., Bogerts B., Perry E.K. (2011). Partial loss of parvalbumin-containing hippocampal interneurons in dementia with Lewy bodies. Neuropathology.

[bb0035] Bhat A.H., Dar K.B., Anees S., Zargar M.A., Masood A., Sofi M.A., Ganie S.A. (2015). Oxidative stress, mitochondrial dysfunction and neurodegenerative diseases; a mechanistic insight. Biomed. Pharmacother..

[bb0040] Born H.A. (2015). Seizures in Alzheimer's disease. Neuroscience.

[bb0045] Briel R.C., McKeith I.G., Barker W.A., Hewitt Y., Perry R.H., Ince P.G., Fairbairn A.F. (1999). EEG findings in dementia with Lewy bodies and Alzheimer’s disease. J. Neurol. Neurosurg. Psychiatry.

[bb0050] Busch D.J., Oliphint P.A., Walsh R.B., Banks S.M., Woods W.S., George J.M., Morgan J.R. (2014). Acute increase of alpha-synuclein inhibits synaptic vesicle recycling evoked during intense stimulation. Mol. Biol. Cell.

[bb0055] Busche M.A., Konnerth A. (2015). Neuronal hyperactivity--a key defect in Alzheimer’s disease?. Bioessays.

[bb0060] Colgin L.L. (2016). Rhythms of the hippocampal network. Nat. Rev. Neurosci..

[bb0065] Costa G., Sisalli M.J., Simola N., Della Notte S., Casu M.A., Serra M., Pinna A., Feliciello A., Annunziato L., Scorziello A., Morelli M. (2020). Gender differences in neurodegeneration, neuroinflammation and Na(+)-Ca(2+) exchangers in the female A53T transgenic mouse model of Parkinson’s disease. Front. Aging Neurosci..

[bb0070] Dougherty S.E., Hollimon J.J., McMeekin L.J., Bohannon A.S., West A.B., Lesort M., Hablitz J.J., Cowell R.M. (2014). Hyperactivity and cortical disinhibition in mice with restricted expression of mutant huntingtin to parvalbumin-positive cells. Neurobiol. Dis..

[bb0075] Ekmark-Lewen S., Lindstrom V., Gumucio A., Ihse E., Behere A., Kahle P.J., Nordstrom E., Eriksson M., Erlandsson A., Bergstrom J., Ingelsson M. (2018). Early fine motor impairment and behavioral dysfunction in (Thy-1)-h[A30P] alpha-synuclein mice. Brain Behav..

[bb0080] Fagerqvist T., Lindstrom V., Nordstrom E., Lord A., Tucker S.M., Su X., Sahlin C., Kasrayan A., Andersson J., Welander H., Nasstrom T., Holmquist M., Schell H., Kahle P.J., Kalimo H., Moller C., Gellerfors P., Lannfelt L., Bergstrom J., Ingelsson M. (2013). Monoclonal antibodies selective for alpha-synuclein oligomers/protofibrils recognize brain pathology in Lewy body disorders and alpha-synuclein transgenic mice with the disease-causing A30P mutation. J. Neurochem..

[bb0085] Fisahn A., Contractor A., Traub R.D., Buhl E.H., Heinemann S.F., McBain C.J. (2004). Distinct roles for the kainate receptor subunits GluR5 and GluR6 in kainate-induced hippocampal gamma oscillations. J. Neurosci..

[bb0090] Flores-Cuadrado A., Ubeda-Banon I., Saiz-Sanchez D., de la Rosa-Prieto C., Martinez-Marcos A. (2016). Hippocampal alpha-synuclein and interneurons in Parkinson’s disease: data from human and mouse models. Mov. Disord..

[bb0095] Freichel C., Neumann M., Ballard T., Muller V., Woolley M., Ozmen L., Borroni E., Kretzschmar H.A., Haass C., Spooren W., Kahle P.J. (2007). Age-dependent cognitive decline and amygdala pathology in alpha-synuclein transgenic mice. Neurobiol. Aging.

[bb0100] Fu C.H., Iascone D.M., Petrof I., Hazra A., Zhang X., Pyfer M.S., Tosi U., Corbett B.F., Cai J., Lee J., Park J., Iacovitti L., Scharfman H.E., Enikolopov G., Chin J. (2019). Early seizure activity accelerates depletion of hippocampal neural stem cells and impairs spatial discrimination in an Alzheimer’s disease model. Cell Rep..

[bb0105] Gomez-Tortosa E., Sanders J.L., Newell K., Hyman B.T. (2001). Cortical neurons expressing calcium binding proteins are spared in dementia with Lewy bodies. Acta Neuropathol..

[bb0110] Gulyas A.I., Buzsaki G., Freund T.F., Hirase H. (2006). Populations of hippocampal inhibitory neurons express different levels of cytochrome c. Eur. J. Neurosci..

[bb0115] Haberman R.P., Branch A., Gallagher M. (2017). Targeting neural hyperactivity as a treatment to stem progression of late-onset Alzheimer’s disease. Neurotherapeutics.

[bb0120] Hall A.M., Throesch B.T., Buckingham S.C., Markwardt S.J., Peng Y., Wang Q., Hoffman D.A., Roberson E.D. (2015). Tau-dependent Kv4.2 depletion and dendritic hyperexcitability in a mouse model of Alzheimer’s disease. J. Neurosci..

[bb0125] Jiang X., Lachance M., Rossignol E. (2016). Involvement of cortical fast-spiking parvalbumin-positive basket cells in epilepsy. Prog. Brain Res..

[bb0130] Kahle P.J., Neumann M., Ozmen L., Muller V., Jacobsen H., Schindzielorz A., Okochi M., Leimer U., van Der Putten H., Probst A., Kremmer E., Kretzschmar H.A., Haass C. (2000). Subcellular localization of wild-type and Parkinson’s disease-associated mutant alpha -synuclein in human and transgenic mouse brain. J. Neurosci..

[bb0135] Kahle P.J., Neumann M., Ozmen L., Muller V., Odoy S., Okamoto N., Jacobsen H., Iwatsubo T., Trojanowski J.Q., Takahashi H., Wakabayashi K., Bogdanovic N., Riederer P., Kretzschmar H.A., Haass C. (2001). Selective insolubility of alpha-synuclein in human Lewy body diseases is recapitulated in a transgenic mouse model. Am. J. Pathol..

[bb0140] Kann O., Huchzermeyer C., Kovacs R., Wirtz S., Schuelke M. (2011). Gamma oscillations in the hippocampus require high complex I gene expression and strong functional performance of mitochondria. Brain.

[bb0145] Kann O., Papageorgiou I.E., Draguhn A. (2014). Highly energized inhibitory interneurons are a central element for information processing in cortical networks. J. Cereb. Blood Flow Metab..

[bb0150] Keane P.C., Hanson P.S., Patterson L., Blain P.G., Hepplewhite P., Khundakar A.A., Judge S.J., Kahle P.J., LeBeau F.E.N., Morris C.M. (2019). Trichloroethylene and its metabolite TaClo lead to degeneration of substantia nigra dopaminergic neurones: effects in wild type and human A30P mutant alpha-synuclein mice. Neurosci. Lett..

[bb0155] Lam A.D., Deck G., Goldman A., Eskandar E.N., Noebels J., Cole A.J. (2017). Silent hippocampal seizures and spikes identified by foramen ovale electrodes in Alzheimer’s disease. Nat. Med..

[bb0160] Lautenschlager J., Kaminski C.F., Kaminski Schierle G.S. (2017). Alpha-Synuclein - regulator of exocytosis, endocytosis, or both?. Trends Cell Biol..

[bb0165] Ludtmann M.H.R., Angelova P.R., Horrocks M.H., Choi M.L., Rodrigues M., Baev A.Y., Berezhnov A.V., Yao Z., Little D., Banushi B., Al-Menhali A.S., Ranasinghe R.T., Whiten D.R., Yapom R., Dolt K.S., Devine M.J., Gissen P., Kunath T., Jaganjac M., Pavlov E.V., Klenerman D., Abramov A.Y., Gandhi S. (2018). α-synuclein oligomers interact with ATP synthase and open the permeability transition pore in Parkinson’s disease. Nat. Commun..

[bb0170] Martin L.J., Semenkow S., Hanaford A., Wong M. (2014). Mitochondrial permeability transition pore regulates Parkinson’s disease development in mutant alpha-synuclein transgenic mice. Neurobiol. Aging.

[bb0175] Masliah E., Rockenstein E., Veinbergs I., Mallory M., Hashimoto M., Takeda A., Sagara Y., Sisk A., Mucke L. (2000). Dopaminergic loss and inclusion body formation in alpha-synuclein mice: implications for neurodegenerative disorders. Science.

[bb0180] McKeith I.G., Boeve B.F., Dickson D.W., Halliday G., Taylor J.P., Weintraub D., Aarsland D., Galvin J., Attems J., Ballard C.G., Bayston A., Beach T.G., Blanc F., Bohnen N., Bonanni L., Bras J., Brundin P., Burn D., Chen-Plotkin A., Duda J.E., El-Agnaf O., Feldman H., Ferman T.J., Ffytche D., Fujishiro H., Galasko D., Goldman J.G., Gomperts S.N., Graff-Radford N.R., Honig L.S., Iranzo A., Kantarci K., Kaufer D., Kukull W., Lee V.M.Y., Leverenz J.B., Lewis S., Lippa C., Lunde A., Masellis M., Masliah E., McLean P., Mollenhauer B., Montine T.J., Moreno E., Mori E., Murray M., O’Brien J.T., Orimo S., Postuma R.B., Ramaswamy S., Ross O.A., Salmon D.P., Singleton A., Taylor A., Thomas A., Tiraboschi P., Toledo J.B., Trojanowski J.Q., Tsuang D., Walker Z., Yamada M., Kosaka K. (2017). Diagnosis and management of dementia with Lewy bodies: fourth consensus report of the DLB consortium. Neurology.

[bb0185] McKeith I.G., Ferman T.J., Thomas A.J., Blanc F., Boeve B.F., Fujishiro H., Kantarci K., Muscio C., O'Brien J.T., Postuma R.B., Aarsland D., Ballard C., Bonanni L., Donaghy P., Emre M., Galvin J.E., Galasko D., Goldman J.G., Gomperts S.N., Honig L.S., Ikeda M., Leverenz J.B., Lewis S.J.G., Marder K.S., Masellis M., Salmon D.P., Taylor J.P., Tsuang D.W., Walker Z., Tiraboschi P. (2020). Research criteria for the diagnosis of prodromal dementia with Lewy bodies. Neurology.

[bb0190] Morris M., Sanchez P.E., Verret L., Beagle A.J., Guo W., Dubal D., Ranasinghe K.G., Koyama A., Ho K., Yu G.Q., Vossel K.A., Mucke L. (2015). Network dysfunction in alpha-synuclein transgenic mice and human Lewy body dementia. Ann. Clin. Transl. Neurol..

[bb0195] Neumann M., Kahle P.J., Giasson B.I., Ozmen L., Borroni E., Spooren W., Muller V., Odoy S., Fujiwara H., Hasegawa M., Iwatsubo T., Trojanowski J.Q., Kretzschmar H.A., Haass C. (2002). Misfolded proteinase K-resistant hyperphosphorylated alpha-synuclein in aged transgenic mice with locomotor deterioration and in human alpha-synucleinopathies. J. Clin. Invest..

[bb0200] Pacheco C.R., Morales C.N., Ramirez A.E., Munoz F.J., Gallegos S.S., Caviedes P.A., Aguayo L.G., Opazo C.M. (2015). Extracellular alpha-synuclein alters synaptic transmission in brain neurons by perforating the neuronal plasma membrane. J. Neurochem..

[bb0205] Palop J.J., Mucke L. (2016). Network abnormalities and interneuron dysfunction in Alzheimer disease. Nat. Rev. Neurosci..

[bb0210] Palop J.J., Chin J., Roberson E.D., Wang J., Thwin M.T., Bien-Ly N., Yoo J., Ho K.O., Yu G.Q., Kreitzer A., Finkbeiner S., Noebels J.L., Mucke L. (2007). Aberrant excitatory neuronal activity and compensatory remodeling of inhibitory hippocampal circuits in mouse models of Alzheimer's disease. Neuron.

[bb0215] Park I.S., Yoo S.W., Lee K.S., Kim J.S. (2014). Epileptic seizure presenting as dementia with Lewy bodies. Gen. Hosp. Psychiatry.

[bb0220] Peters S.T., Fahrenkopf A., Choquette J.M., Vermilyea S.C., Lee M.K., Vossel K. (2020). Ablating tau reduces hyperexcitability and moderates electroencephalographic slowing in transgenic mice expressing A53T human α-Synuclein. Front. Neurol..

[bb0225] Price D.L., Rockenstein E., Ubhi K., Phung V., MacLean-Lewis N., Askay D., Cartier A., Spencer B., Patrick C., Desplats P., Ellisman M.H., Masliah E. (2010). Alterations in mGluR5 expression and signaling in Lewy body disease and in transgenic models of alpha-synucleinopathy--implications for excitotoxicity. PLoS One.

[bb0230] Procaccini C., Maksimovic M., Aitta-Aho T., Korpi E.R., Linden A.M. (2013). Reversal of novelty-induced hyperlocomotion and hippocampal c-Fos expression in GluA1 knockout male mice by the mGluR2/3 agonist LY354740. Neuroscience.

[bb0235] Reeve A.K., Ludtmann M.H., Angelova P.R., Simcox E.M., Horrocks M.H., Klenerman D., Gandhi S., Turnbull D.M., Abramov A.Y. (2015). Aggregated alpha-synuclein and complex I deficiency: exploration of their relationship in differentiated neurons. Cell Death Dis..

[bb0240] Robson E., Tweedy C., Manzanza N., Taylor J.P., Atkinson P., Randall F., Reeve A., Clowry G.J., LeBeau F.E.N. (2018). Impaired fast network oscillations and mitochondrial dysfunction in a mouse model of alpha-synucleinopathy (A30P). Neuroscience.

[bb0245] Rockenstein E., Mallory M., Hashimoto M., Song D., Shults C.W., Lang I., Masliah E. (2002). Differential neuropathological alterations in transgenic mice expressing alpha-synuclein from the platelet-derived growth factor and Thy-1 promoters. J. Neurosci. Res..

[bb0250] Sanchez P.E., Zhu L., Verret L., Vossel K.A., Orr A.G., Cirrito J.R., Devidze N., Ho K., Yu G.Q., Palop J.J., Mucke L. (2012). Levetiracetam suppresses neuronal network dysfunction and reverses synaptic and cognitive deficits in an Alzheimer’s disease model. Proc. Natl. Acad. Sci. U. S. A..

[bb0255] Scharfman H.E., MacLusky N.J. (2014). Sex differences in the neurobiology of epilepsy: a preclinical perspective. Neurobiol. Dis..

[bb0260] Schell H., Hasegawa T., Neumann M., Kahle P.J. (2009). Nuclear and neuritic distribution of serine-129 phosphorylated alpha-synuclein in transgenic mice. Neuroscience.

[bb0265] Singh B., Covelo A., Martell-Martinez H., Nanclares C., Sherman M.A., Okematti E., Meints J., Teravskis P.J., Gallardo C., Savonenko A.V., Benneyworth M.A., Lesne S.E., Liao D., Araque A., Lee M.K. (2019). Tau is required for progressive synaptic and memory deficits in a transgenic mouse model of alpha-synucleinopathy. Acta Neuropathol..

[bb0270] Spillantini M.G., Schmidt M.L., Lee V.M., Trojanowski J.Q., Jakes R., Goedert M. (1997). Alpha-synuclein in Lewy bodies. Nature.

[bb0275] Stylianou M., Zaaimi B., Thomas A., Taylor J.P., LeBeau F.E.N. (2020). Early disruption of cortical sleep-related oscillations in a mouse model of dementia with Lewy bodies (DLB) expressing human mutant (A30P) alpha-synuclein. Front. Neurosci..

[bb0280] Sugino K., Hempel C.M., Miller M.N., Hattox A.M., Shapiro P., Wu C., Huang Z.J., Nelson S.B. (2006). Molecular taxonomy of major neuronal classes in the adult mouse forebrain. Nat. Neurosci..

[bb0285] Ukai K., Fujishiro H., Watanabe M., Kosaka K., Ozaki N. (2017). Similarity of symptoms between transient epileptic amnesia and Lewy body disease. Psychogeriatrics.

[bb0290] Unger E.L., Eve D.J., Perez X.A., Reichenbach D.K., Xu Y., Lee M.K., Andrews A.M. (2006). Locomotor hyperactivity and alterations in dopamine neurotransmission are associated with overexpression of A53T mutant human alpha-synuclein in mice. Neurobiol. Dis..

[bb0295] Vossel K.A., Ranasinghe K.G., Beagle A.J., Mizuiri D., Honma S.M., Dowling A.F., Darwish S.M., Van Berlo V., Barnes D.E., Mantle M., Karydas A.M., Coppola G., Roberson E.D., Miller B.L., Garcia P.A., Kirsch H.E., Mucke L., Nagarajan S.S. (2016). Incidence and impact of subclinical epileptiform activity in Alzheimer’s disease. Ann. Neurol..

[bb0300] Vossel K.A., Tartaglia M.C., Nygaard H.B., Zeman A.Z., Miller B.L. (2017). Epileptic activity in Alzheimer’s disease: causes and clinical relevance. Lancet Neurol..

[bb0305] Whittaker R.G., Turnbull D.M., Whittington M.A., Cunningham M.O. (2011). Impaired mitochondrial function abolishes gamma oscillations in the hippocampus through an effect on fast-spiking interneurons. Brain.

[bb0310] Whittington M.A., Cunningham M.O., LeBeau F.E., Racca C., Traub R.D. (2011). Multiple origins of the cortical gamma rhythm. Dev. Neurobiol..

[bb0315] Wu N., Joshi P.R., Cepeda C., Masliah E., Levine M.S. (2010). Alpha-synuclein overexpression in mice alters synaptic communication in the corticostriatal pathway. J. Neurosci. Res..

[bb0320] Yamada K., Iwatsubo T. (2018). Extracellular alpha-synuclein levels are regulated by neuronal activity. Mol. Neurodegener..

[bb0325] Yassa M.A., Stark S.M., Bakker A., Albert M.S., Gallagher M., Stark C.E. (2010). High-resolution structural and functional MRI of hippocampal CA3 and dentate gyrus in patients with amnestic mild cognitive impairment. Neuroimage.

[bb0330] Yoshikawa M., Soeda Y., Michikawa M., Almeida O.F.X., Takashima A. (2018). Tau depletion in APP transgenic mice attenuates task-related hyperactivation of the Hippocampus and differentially influences locomotor activity and spatial memory. Front. Neurosci..

[bb0335] Zhang W., Zhang L., Liang B., Schroeder D., Zhang Z.W., Cox G.A., Li Y., Lin D.T. (2016). Hyperactive somatostatin interneurons contribute to excitotoxicity in neurodegenerative disorders. Nat. Neurosci..

